# Qualitative Assessment of Off-Gassing of Compounds from Field-Contaminated Firefighter Jackets with Varied Air Exposure Time Intervals Using Headspace GC-MS

**DOI:** 10.3390/textiles3020016

**Published:** 2023-06-07

**Authors:** Arjunsing Girase, Adhiraj Shinde, Robert Bryan Ormond

**Affiliations:** Textile Protection and Comfort Center, Wilson College of Textiles, North Carolina State University, Raleigh, NC 27606, USA

**Keywords:** off-gassing, firefighters, headspace sampling, GC-MS

## Abstract

Firefighters are exposed to a complex mix of volatile and semi-volatile compounds from burning construction materials, consumer products, and other elements during fire suppression and rescue. These compounds can be absorbed onto the gear worn by firefighters and, depending on their volatility, can be released from the gear under different conditions. Few studies have focused on the off-gassing of toxic compounds from firefighters’ gear, particularly in terms of qualitative analysis methods. This study introduces a novel qualitative analysis method using headspace gas chromatography–mass spectrometry (HS-GC-MS) to assess off-gassing from field-contaminated jackets at regular intervals. Our findings show that certain compounds, such as acetic acid and di-ethyl-hexyl-phthalate (DEHP), remained present even after the gear were allowed to air out for 48 h. The persistent off-gassing of chemicals, even under ambient conditions, raises concerns about potential hazards that could pose risks for personnel in the vicinity of contaminated gear, including inside fire stations. The implications of these findings extend beyond fire stations and may have significant public health implications for firefighters who are repeatedly exposed to these compounds over time.

## 1. Introduction

Firefighters are exposed to a range of hazardous substances during their work, including toxic chemicals released during fire-suppression activities. These chemicals can pose serious health risks to firefighters, including increasing their risk of cancer and respiratory disease. Protective gear is essential for reducing the risk of exposure to these toxic substances; however, the gear itself can also adsorb hazardous chemicals. Depending on the volatility of the chemicals, they can subsequently be off-gassed and released into the environment, potentially exposing firefighters and others to toxic fumes. Previous research has investigated the off-gassing of volatile organic compounds (VOCs) from firefighters’ protective equipment, detecting over 64 different compounds [1]. Despite the potential hazards posed by contaminated gear, limited research has been conducted on the off-gassing of chemicals from firefighter gear. Smoke from fires is composed of a combination of suspended liquid and solid particulate matters, as well as gases and vapors that arise from the combustion or pyrolysis of materials. In a previous study, a single training session with French firefighters exposed them to Benzo[a]pyrene, which is a known carcinogen [2]. Similarly, fluorene and pyrene were found in the urine of firefighters after a session of controlled residential fire, which showed that PAHs were absorbed into the body [3]. The characterization of firefighter smoke exposure showed the presence of PAHs and phthalates [4]. An analysis of unused and used jackets from the Cincinnati fire department showed the presence of di-ethyl-hexyl phthalate (DEHP) [5].

Decontamination practices are ineffective in removing contaminants from firefighter gear [6–8]. Most of the studies have focused on identifying contaminants present on firefighter gear, assessing the presence of diverse volatile organic compounds in the air during fire incidents, or evaluating the effectiveness of decontamination procedures [9–12]. However, they have not analyzed the off-gassing of chemicals from PPE. In general, these studies have focused on contaminants with low volatilities, although there are a few exceptions that investigated some of the lower-molecular-weight polycyclic aromatic hydrocarbons (PAHs).

Headspace sampling (HS) as an air sampling technique has gained impetus due to its ease of operation and simple sample preparation. This technique can practically be used to analyze the off-gassing of chemicals from any solid or liquid matrix. The headspace sampler is an instrument used to heat solid/liquid samples in a glass crimp-top vial, typically in the temperature range of 35 °C to 200 °C. The vial can then be placed in an oven and equilibrated for a specific time. The vapors generated from the samples get collected in the headspace between the samples and the vial cap. A needle is inserted into the vial and transfers the vapors to the heated HS transfer line via a heated loop system. The vapors are then directly injected into the gas chromatograph (GC) inlet through the heated transfer line. This process ensures that there is no compound loss and that no vapor condensation occurs inside the machine parts. The headspace gas chromatography–mass spectrometry system has the capability to analyze the off-gassing of compounds from a substrate, provided appropriate calibration standards are available [12]. The headspace sampling technique has been used in a variety of applications, such as measuring acetaldehyde levels to detect alcohol consumption [13], measuring volatile compounds from cow’s milk [14], measuring halogenated VOCs from industrial groundwater [15], and many others [16–21].

Specifically, this study employed the HS-GC-MS technique to measure the levels of off-gassing of fireground contaminants from field-contaminated firefighter turnout jackets at 200 °C. A structure containing various materials, such as electronics, papers, and plastics, that a regular house would have was set on fire by the local fire department for training purposes. For this study, two jackets were hung in one of the rooms and exposed to the smoke that was caused by the fire. Subsequently, after the fire was extinguished, samples from the jackets were taken at different time intervals and analyzed using the headspace technique for levels of various volatile and semi-volatile compounds found to be off-gassing. This research focused on the thermal extraction/off-gassing of certain compounds present in the fabrics even after they were allowed to be exposed to air for fixed intervals. The findings are important since knowing the types of compounds off-gassing from firefighter gear will enable us to better protect firefighters from occupational exposure related to their profession. The possible off-gassing of highly volatile compounds from firefighter gear at ambient conditions, such as inside a fire station, could be hazardous to the health of firefighters. This is because the personnel inside fire stations are typically not wearing respiratory protection equipment, and the presence of toxic compounds in the ambient atmosphere could pose a respiratory hazard.

The findings of this study are critical for better understanding the potential health risks associated with the off-gassing of contaminants from firefighter gear. This study will help in addressing the significant research gap in the field since existing studies have primarily focused on identifying contaminants on gear surfaces or evaluating decontamination procedures [22,23]. There is a lack of comprehensive understanding regarding the extent to which contaminated gear off-gas and pose a hazard to firefighters. Knowledge of the types of compounds off-gassing from contaminated gear can help develop strategies to better protect firefighters from occupational exposure related to their profession. The possibility of highly volatile compounds off-gassing from firefighter gear at ambient conditions, such as inside a fire station, could pose a significant respiratory hazard for personnel who are not wearing respiratory protection equipment. Therefore, this study aims to address this research gap by investigating the off-gassing of contaminants from field-contaminated firefighter jackets, using the innovative approach of headspace gas chromatography–mass spectrometry (HS-GC-MS) to qualitatively analyze the volatile and semi-volatile compounds released from the gear over different time intervals of air exposure.

## 2. Materials and Methods

### 2.1. Materials

A used and an unused turnout jacket were obtained from the Raleigh training facility. The 20 mL headspace glass vials, crimp tops, crimper, and de-crimper were purchased from Agilent Technologies.

### 2.2. Sample Collection

A live fire training was conducted in a building scheduled for demolition. The used and unused turnout jackets were hung in the room to be burned. The contents of the building, including furniture, magazines, electronics, and plastics, were not removed to simulate realistic fire exposure. The jackets were allowed to hang in the room until the fire was extinguished with pressurized water.

After the jackets were removed from the burned room, one large piece of fabric, measuring approximately 30 cm × 30 cm, was cut from the outer shell layer of the used and unused jackets, respectively. Three replicate fabric samples measuring 5 cm × 5 cm were then cut from each jacket and placed in 20 mL crimp-top glass vials at 0 h, 0.5 h, 1 h, 4 h, and 48 h after the jackets were removed from the burned room. The vials were crimped as soon as the fabric samples were placed inside to prevent any gases from escaping into the atmosphere.

### 2.3. Headspace-GC-MS Chromatographic Method

Chromatographic analysis was conducted using the split mode with a split ratio of 10:1 to ensure accurate and sensitive chromatographic analysis. The column used in the GC was an Agilent EPA 8270D fused silica capillary column (30 m × 0.25 mm × 0.25 μm). An Agilent 5190–3136 UI splitless single taper with a glass wool liner was used in the inlet for injection. The injection volume was 1 μL, the injection temperature was kept at 250 °C, and the helium flow rate was 1.2 mL/min. All of these conditions facilitated proper sample introduction.

The vials containing the fabric samples were loaded into the 7697A Agilent headspace sampler connected to a 7890B GC and a 5977B MS. The equilibration temperature was set at 200 °C and the equilibration time was set for 30 min to detect contaminants. The chromatographic conditions were set as follows: the oven gradient was set to begin at 40 °C, increased to 280 °C for 1 min at a rate of 10 °C/min, and further increased to 300 °C at 5 °C/min for 1 min. The total run time was 30 min. The MS transfer line was kept at 280 °C throughout the run to mitigate any loss of volatile compounds. The MS quadrupole temperature was maintained at 230 °C and the ion source temperature was kept at 150 °C. A gain factor of 1.00 was used. The analysis was conducted in the scan mode (35–550 amu) using electron ionization (EI) with an energy of 70 eV.

### 2.4. Quality Check

A custom standard containing DEHP in liquid form was run through the headspace to confirm the compound’s retention time. The details of the specific compounds and analysis are provided by Shinde et al. [24].

## 3. Results

### 3.1. Thermal Extraction of Field-Contaminated Jackets Using Headspace GC at 200 °C for 30 min

**Figure 1.**
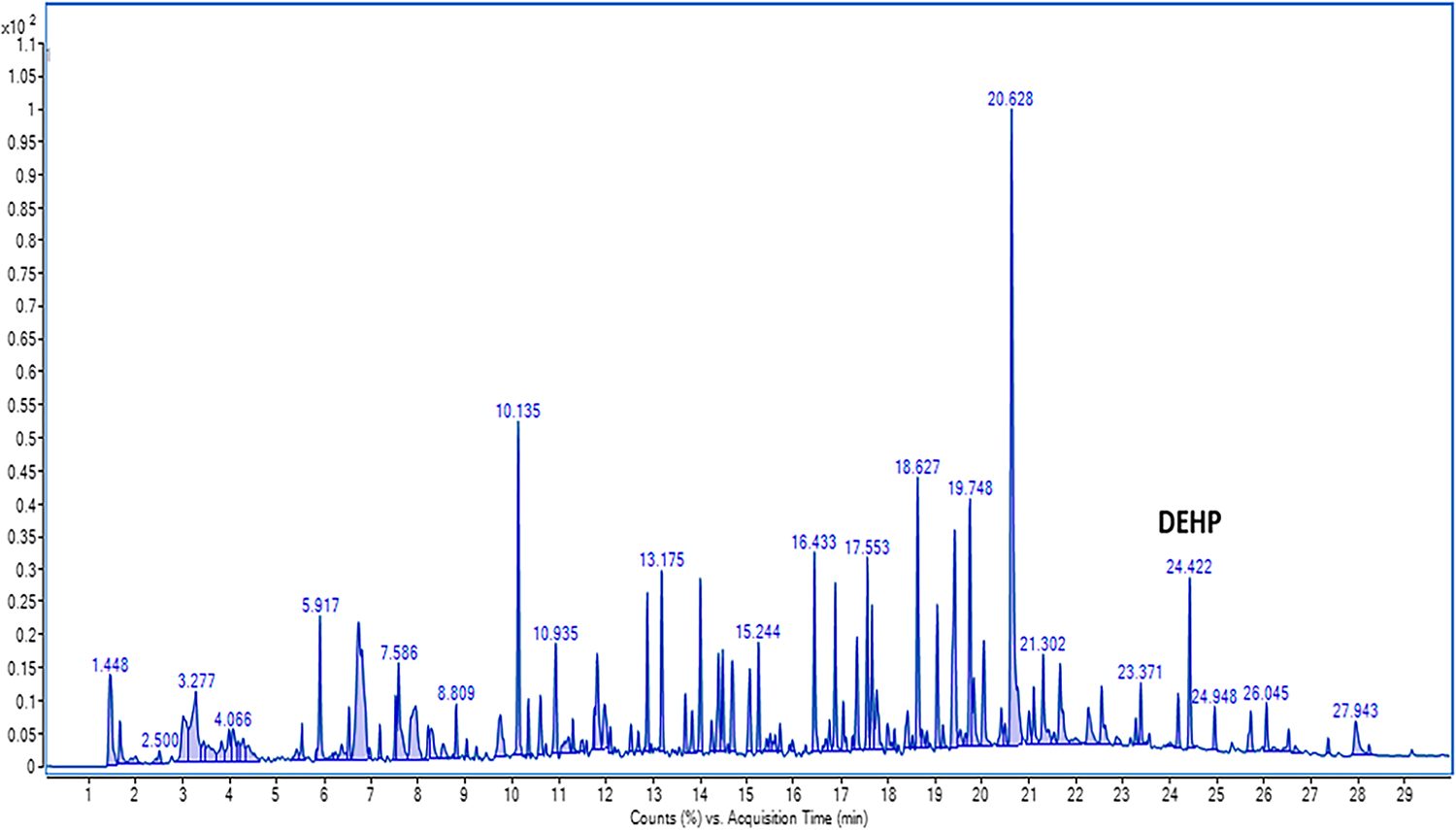
The ‘0-h’ air exposure sample’s chromatogram for compounds off-gassing from the used turnout jacket using headspace-GC-MS at 200 °C for 30 min.

**Figure 2.**
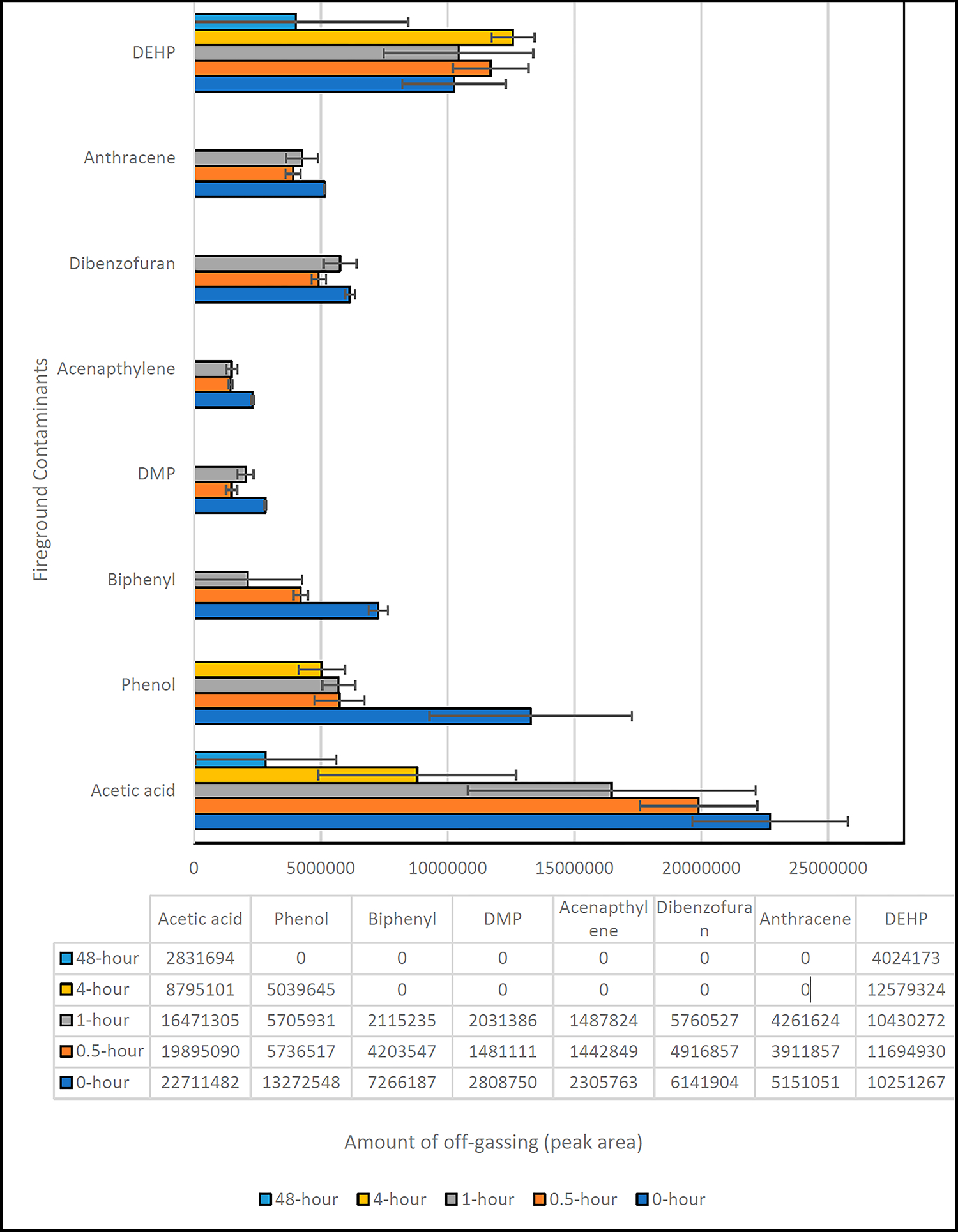
Amounts of off-gassing of compounds from the used turnout jacket.

The aim of this study was to understand the type of compounds that off-gas from a used gear after a live burn. Another objective was to understand the effect of air exposure on the amounts of compounds off-gassing from the outer shell materials. A temperature of 200 °C with an equilibration time of 30 min was the best-suited condition for the thermal extraction of outer shell materials. Figure 1 shows the chromatogram of the headspace GC analysis of the field-contaminated firefighter fabric samples conducted at 200 °C for 30 min while Figure 2 shows the amount of compounds off-gassed. The samples are termed ‘0-h’ exposure, meaning that the sample was immediately placed inside the HS vial after the exposed jacket was removed from the burning building.

**Figure 3.**
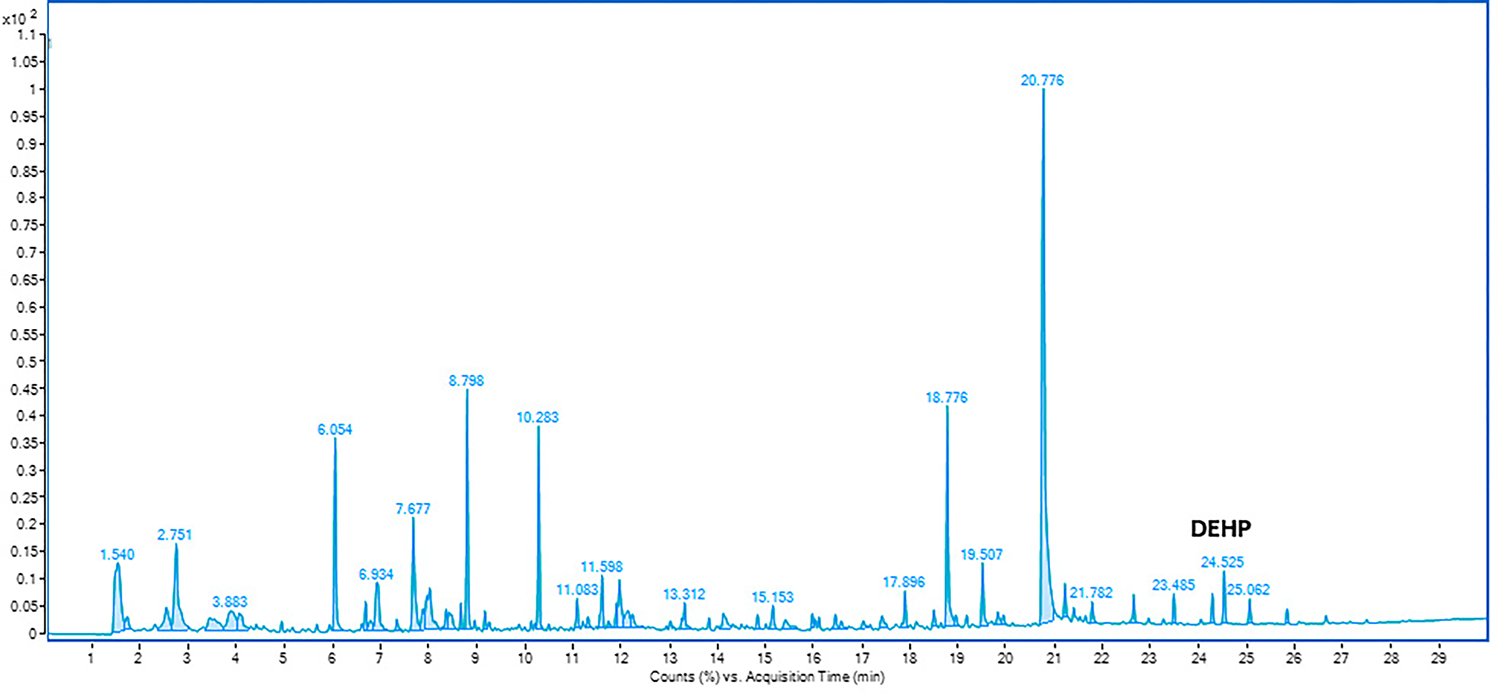
The ‘0-h’ air exposure chromatogram for compounds off-gassing from the unused turnout jacket using headspace-GC-MS at 200 °C for 30 min.

As seen in Figures 1 and 3, several peaks are generated in the chromatogram. When analyzing each peak using the NIST 17 Mass Spectral library, the peaks having a minimum percent match of 50% and above were chosen. Additionally, the peaks having a peak height significantly above the baseline were considered. Figure 1 shows the amounts of off-gassing of compounds from the used field-contaminated firefighting gear. The values of the compounds off-gassing are the average amounts (peak areas) taken from the three replicate fabric samples analyzed.

### 3.2. Analysis of the Used Turnout Jacket

The fabric samples taken from the used turnout jacket were analyzed for off-gassing of compounds using the headspace GC at a temperature of 200 °C for an equilibration time of 30 min. The chromatogram shown in Figure 1 displays the ‘0-h’ air exposure sample, which was obtained immediately after removing the jacket from the burning building. This sample represents the initial contamination level of the jacket following exposure to contaminants in the burning building. It is important to note that this figure is only a snapshot of the off-gassing behavior at the beginning of our study. The overall study duration extended beyond this immediate time point, allowing us to examine the off-gassing process over a four-hour period. By analyzing subsequent samples at different time intervals, we aimed to capture the dynamic nature of off-gassing and gain insights into the persistence and release of volatile and semi-volatile compounds from the contaminated gear. This approach provides a comprehensive understanding of the off-gassing behavior beyond the initial exposure and can shed light on the potential risks associated with prolonged exposure to firefighter gear.

The air exposure times compared in this study, as presented in Figure 2, were 0 h, 0.5 h, 1 h, 4 h, and 48 h. Figure 2 illustrates a discernible trend for acetic acid—the quantity detected diminished as the air exposure time increased. This result aligns with acetic acid’s high volatility, which would cause it to off-gas when exposed to air rapidly.

Phenol’s trend is particularly noteworthy. In the first 30 min of air exposure, a drastic decrease in off-gassing was observed, which then plateaued for the subsequent 30 min. A minor decrease followed at the 4 h mark, and by 48 h, no phenol could be detected. This trend corroborates with the inherent volatile nature of phenol, implying its rapid off-gassing from the outer shell fabrics when exposed to air.

The biphenyl trend observed in Figure 2 presents an interesting scenario. It exhibited a consistent decrease in off-gassing from 0 h to 1 h, and it became undetectable at the 4 h and 48 h points. An important observation in this case is the broad error bars at the 1 h mark, indicating possible variation in the presence of biphenyl across the replicates.

For acenaphthylene, dibenzofuran, and anthracene, an atypical off-gassing trend is discernible in Figure 2. These compounds showed a minor decrease from 0 h to 0.5 h, an increase at the 1 h mark, and became undetectable at both the 4 h and 48 h marks. This pattern might suggest a complex off-gassing process for these compounds and merit further investigation.

The off-gassing pattern of DEHP displays considerable variability over the different air exposure times. Notably, despite its semi-volatility and high boiling point of 384 °C, DEHP was detectable even at the 48 h exposure time. However, the extensive error bar, which spans the entire compound bar, indicates a degree of inconsistency in the presence of DEHP across different replicates. This observation might suggest a complex interaction between DEHP and the material of the firefighter jacket, which could be further investigated.

Finally, Figure 2 shows dimethyl phthalate (DMP) being detected from 0 h until 1 h, but it is absent after 1 h of air exposure. This implies a rapid off-gassing process for DMP in the initial stages of air exposure, which completes within the first hour. This result provides a crucial insight into the behavior of DMP under air exposure conditions.

### 3.3. Analysis of Unused Turnout Jacket

The fabric samples taken from the unused turnout jacket were analyzed for off-gassing of compounds using the headspace GC at a temperature of 200 °C for an equilibration time of 30 min. The chromatogram shown in Figure 3 shows a number of peaks, each of which represents a different compound. It can be inferred from the chromatogram that the unused turnout jacket was exposed to a lot of contaminants, which were off-gassed during analysis. Comparing Figure 1 to Figure 3, the used turnout jacket shows much higher contamination (higher number of peaks) than the unused turnout jacket. It seems reasonable that the used turnout jacket had a higher contamination since it was possibly exposed to contaminants at multiple instances throughout its service life.

**Figure 4.**
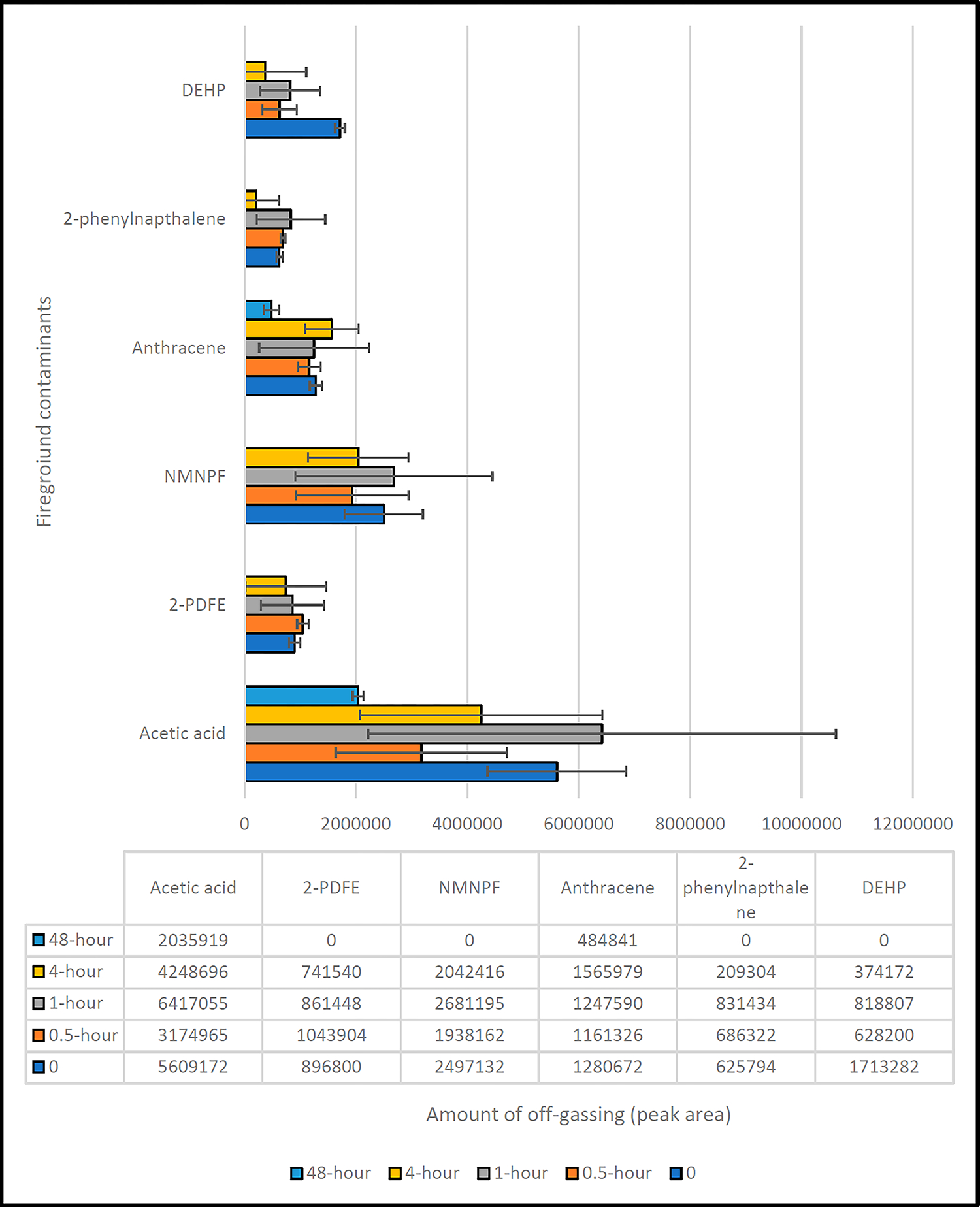
Amounts of off-gassing of compounds from the unused turnout jacket.

Figure 4 presents the off-gassing quantities of various compounds from the field-contaminated firefighting gear over different air exposure times: 0 h, 0.5 h, 1 h, 4 h, and 48 h.

The trend for acetic acid depicted in Figure 4 is particularly intriguing. The quantity of acetic acid off-gassing declined from 0 h to 0.5 h, which is in line with its high volatility, but then surprisingly increased from 0.5 h to 1 h. This unexpected rise contradicts the expected behavior of a volatile compound and warrants further investigation. The wide error bar at the 1 h mark indicates substantial variability across all replicates, suggesting a complex interaction between acetic acid and the gear material. The quantity then reduced again for the 4 h and 48 h samples, following the expected trend.

Figure 4 also illustrates the trend for 2-Propenoic acid-heptadecafluorodecyl ester (2-PDFE). The off-gassing quantity remained fairly consistent from 0 h to 4 h, suggesting a gradual release of the compound over time. However, it was absent at the 48 h mark, indicating a complete off-gassing within this period.

The off-gassing patterns of N-methyl-N-phenyl-formamide (NMNPF), anthracene, and 2-phenyl naphthalene presented in Figure 4 do not show a distinct correlation with time. The quantities off-gassing varied randomly but generally remained within the same range, suggesting that these compounds might off-gas at a relatively constant rate over time or might exhibit a complex off-gassing process.

Lastly, the trend for DEHP in Figure 4 shows a decrease from 0 h to 0.5 h, and a slight increase from 0.5 h to 1 h, followed by a reduction at 4 h and an absence at 48 h. This trend may suggest an initial rapid off-gassing, followed by a slower release rate, and finally a complete off-gassing by 48 h.

In conclusion, the trends observed in Figure 4 highlight the complex off-gassing behaviors of various compounds from the field-contaminated firefighting gear. These findings provide valuable insights for understanding the potential health risks associated with contaminated firefighting gear and inform strategies to mitigate these risks.

## 4. Conclusions

The headspace sampler-GC-MS setup was tested, and a suitable method was developed to analyze the off-gassing of compounds from field-contaminated firefighter gear. The analysis of the field-contaminated gear using the HS-GC-MS at 200 °C for 30 min at various times of air exposure showed that certain compounds do off-gas at a higher rate than others. This study focused on the amounts of compounds readily available to off-gas after fixed time intervals of air exposure. This meant that some quantities of these compounds might have been off-gassed while they were allowed to be exposed to air, but the remaining quantities were still present in the fabrics and did off-gas even after 48 h. Focusing on the 48 h samples, they showed a decrease in off-gassing levels for most compounds, with a higher level of off-gassing for the 0 h samples. This indicates that if a gear is stored after exposure, certain compounds remain present even after 48 h. A contaminated gear, when stored in the engine bay of a fire station, could possibly off-gas certain compounds and create a toxic environment for firefighters. Based on the analysis, volatile compounds having lower boiling points, such as acetic acid, were observed to off-gas significantly even when the contaminated gear was tested at 48 h. This poses a health hazard to firefighters present in a fire station in the absence of respiratory protection equipment.

## 5. Future Work

Using a specialized headspace GC-MS method, this study provided a qualitative assessment of off-gassing from firefighter turnout gear. However, there is a need for a quantitative assessment of off-gassed compounds to understand the occupational risks associated with firefighter turnout gear. The developed headspace GC-MS method can be further improved by developing standard calibration samples for selected compounds. This will enable a quantitative assessment of off-gassed compounds and provide valuable insights for developing strategies to mitigate the risks associated with contaminated gear. In a behavioral study, firefighters showed positive approach and norms toward showering after fire exposure. Still, it was stated that decontamination practices, such as the use of wipes and on-scene decontamination, occurred less frequently, which implies that assessing the off-gassing of chemicals is even more important [25]. The presence of chemical compounds further emphasizes the need to incorporate various decontamination strategies, such as preliminary exposure reduction and conventional washing, to mitigate chronic exposures. It is also advisable for firefighters to bag their contaminated gear after use and keep it separate from areas where firefighters could breathe in chemicals directly.

The limitation of this study is the lack of information on the history of the jackets used. The jackets were obtained from a local fire department, and the history of the jackets, such as the number of fires they were exposed to and the types of fires, was unknown. Future studies could consider collecting this information to better understand the effect of exposure history on the off-gassing of contaminants.

In addition to the turnout gear, the developed headspace method can assess off-gassing from other ensemble elements, such as trousers, gloves, and hoods. The method could also be used to study the off-gassing of volatile compounds after various decontamination studies, such as liquid CO_2_, which has been proven more effective than conventional washing [26].

Moreover, the developed method would be useful to qualitatively and quantitatively assess off-gassing from the gear of wildland firefighters, which has a different exposure profile than structural firefighting. Such studies can potentially impact firefighters’ health and safety significantly.

Future studies could also include the development of effective strategies to mitigate the risks associated with contaminated gear. By understanding the types and quantities of compounds off-gassing from contaminated gear, appropriate mitigation strategies can be developed to ensure the safety of firefighters.

## Data Availability

All data supporting the findings of this study are available within the article.
